# Supplementary material for: Early adulthood socioeconomic trajectories contribute to inequalities in adult diet quality, independent of childhood and adulthood socioeconomic position
Source: J Epidemiol Community Health. 2024 Sep 18;79(2):e222472. doi: 10.1136/jech-2024-222472 (PMC11874354; doi:10.1136/jech-2024-222472)
Supplement: online supplemental file 1 [file jech-79-2-s001.pdf]

Supplementary information

Supplementary methods

Energy adjustment for food intake: further details of the residual method

For the evaluation of diet quality independent of the total diet quantity, we uniformly adjusted energy intake to 2500 kcal/day (10.46 MJ/day), using the residual method. In this method, the energy-adjusted food intake is the residual from the regression model in which total energy intake in the survey day is the independent variable and absolute food intake is the dependent variable. The residual is therefore an estimate of food intake uncorrelated with total energy intake and directly related to overall variation in food choice and composition. The estimated terms in the regression model (formula 1) are used to calculate the predicted food intake with total energy intake set at 2500 kcal/day.

$EFI = \alpha + \beta E + \sigma$ (1),

in which EFI represents energy-adjusted food intake, E represents total energy intake, α, β and σ are respectively the constant term, coefficient and residual error of the regression model.

Causal mediation analysis: further details of the effect estimation

In the causal mediation analysis, average direct effect (ADE; formula 1) evaluated the difference in the predicted value of PyrMDS when a participant switched from the Continued Education class to another SET class, while the mediator, household income or neighbourhood deprivation, was held constant at the predicted value that would take for a given SET class. Average causal mediating effect (ACME; formula 2) evaluated how the predicted value of PyrMDS would change when the mediator took the predicted value at the Continued Education class versus at another SET class, while a participant's assignation to a SET class remained unchanged.

$$ADE = Y_i\big(1, M_i(x)\big) - Y_i\big(0, M_i(x)\big) \quad (1)$$

$$ACME = Y_i\big(x, M_i(1)\big) - Y_i\big(x, M_i(0)\big) \quad (2),$$

where $M_i(x)$ represents the predicted value of a mediator M for unit i under the exposure status x, and $Y_i\big(x, M_i(x)\big)$ represents the predicted value of the outcome that would result if the exposure and mediator take the exposure status x (1 as the treatment category and 0 as the reference category).

Causal mediation analysis: further details of the sensitivity analysis

To examine the robustness of the estimated mediation effects, we conducted three supplementary analyses. First, we tested possible biases from mediator-outcome confounders. Based on the assumption of sequential ignorability[1], we varied the values

[1] Source: Imai, K., Keele, L., Tingley, D. A general approach to causal mediation analysis. Psychological methods 2010, 15(4), 309.

of correlation coefficients between the residuals of the mediator and outcome regressions to observe changes in the estimated ACMEs. Second, we excluded participants of the Economically Inactive class from the mediation analysis, considering that this class was different from the remaining occupation-driven SET classes, and showed a large variance in the predicted mean PyrMDS in the OLS regression model. Third, we refitted the OLS regression and causal mediation models based on the participants who have data on the diet outcome (n=5943).

Supplementary figures

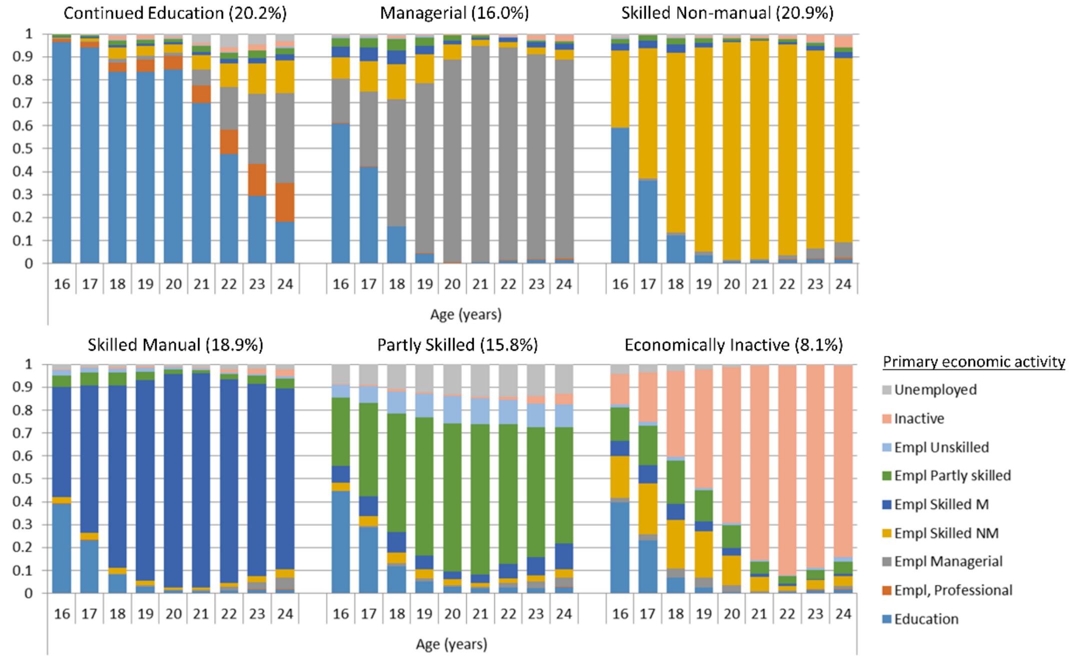

The six-class solution has an entropy of 0.97, indicating a high classification accuracy.

Figure S1. The six socioeconomic trajectory classes, showing response probabilities for participation in different educational and economic activities at each year of age (Winpenny et al., 2021)

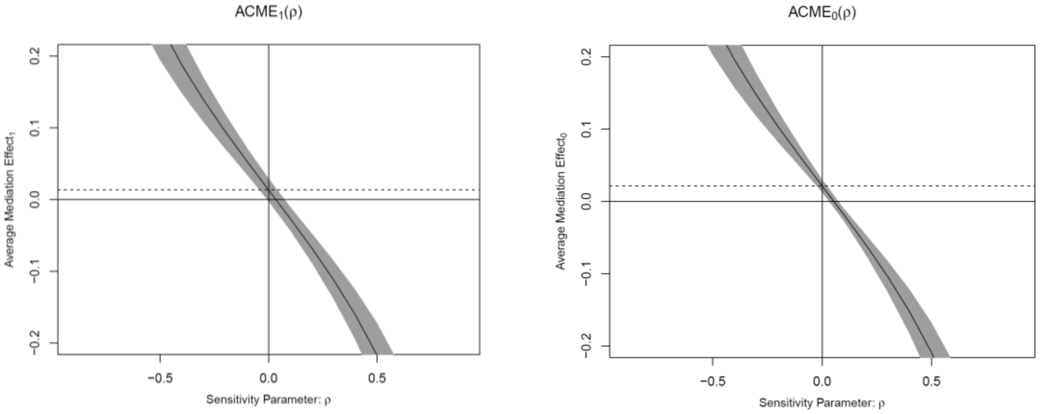

(a) the Continued Education class (b) the remaining SET classes

Figure S2. A sensitivity test for the mediation of neighbourhood deprivation in the association between early adulthood socioeconomic trajectories and adult diet quality, as a function of the correlation between the residuals of the mediator and outcome regression models

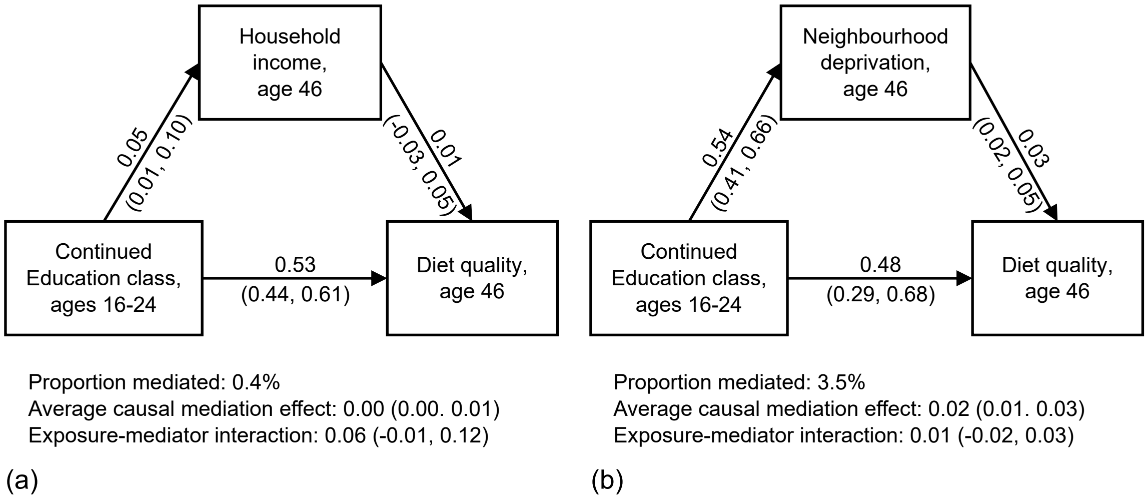

Results are shown in β (95% CI), and are adjusted for covariates.

Figure S3. The causal mediation analysis of the association between early adulthood socioeconomic trajectories and adult diet quality via (a) household income and (b) neighbourhood deprivation at age 46 years, after excluding the participants assigned to the Economically Inactive class (n=11416)

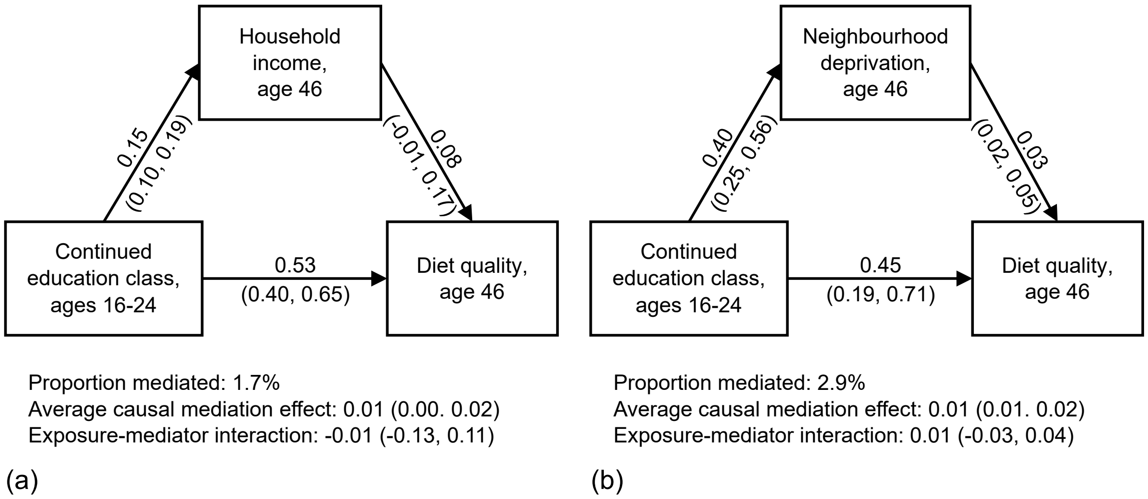

Results are shown in β (95% CI), and are adjusted for covariates.

Figure S4. The causal mediation analysis of the association between early adulthood socioeconomic trajectories and adult diet quality via (a) household income and (b) neighbourhood deprivation at age 46 years, based on participants who have data on the diet outcome (n=5943)

Supplementary tables

Table S1. The pyramid-based Mediterranean diet score (PyrMDS), food components and food items at age 16 and 46 years for the BCS70 participants

**Food components**	**Food items at 16 years**	**Food items at 46 years**	**PyrMDS (0-15)**	
			Serving required for the score of 0	Serving required for the score of 1
Vegetables	fresh and frozen vegetables, canned and processed vegetables, tomatoes, green vegetables, carrots, salad vegetables	raw salad, green leafy/cabbages, root vegetables, tomatoes, allium vegetables, other vegetables (including mushrooms), fruiting and mixed vegetables, vegetable side dishes, vegetable dips	0/d	≥6/d
Legumes	peas, baked beans	meat substitutes – soy, peas/sweetcorn, legumes & pulses	0/wk	≥2/wk
Fruits	fresh fruit, not citrus/apple/pear, canned sweetened fruit, citrus fruits, apple	citrus, berries, apples & pears, other fruit, dried fruit, stewed fruit	0/d	3-6/d
Nuts	nuts	salted nuts & seeds, unsalted nuts & seeds	0/d	1-2/d
Cereals	white bread, wholemeal bread, brown wheatgerm granary bread, other breads, high fibre breakfast cereals, other breakfast cereals, unsweetened, sweetened breakfast cereals, pasta rice cereals	white bread, wholemeal bread, mixed (50/50), brown & seeded, other bread, bran cereal, biscuit cereal, oat cereal (non sugar), oat cereal (sugar), muesli, other cereal (sugar), white pasta & rice, wholemeal pasta, brown rice & other wholegrains, grain dishes - added fat	0/d	3-6/d
Dairy	whole milk, semi-skimmed milk, skimmed milk, other milk and cream, yoghurt, cottage cheese, cheese	whole milk, semiskimmed milk, skimmed milk, rice/oat milk, soy milk, full fat yogurt, low fat yogurt, high fat cheese, medium and low fat cheese	0/d	1.5-2.5/d
Fish	fish, fish dishes	white fish & tinned tuna, shellfish, oily fish, breaded/battered fish	0/wk	≥2/wk
Red meats	beef, lamb and mutton, pork, offal and dishes, meat dishes	pork, beef, lamb, other meat & offal	≥4/wk	<2/wk
Processed meats	meat pies, burgers & kebabs, sausages, bacon & ham, canned meat	processed meat, breaded/battered chicken	≥2/wk	≤1/wk
White meats	coated chicken products	poultry	0/wk	1.5-2.5/wk
Eggs	eggs, egg and cheese dishes	egg & egg dishes	0/wk	2-4/wk
Potatoes	potatoes not fried, fried, roast potato not chips	potatoes/sweet potatoes (baked/boiled), mashed potatoes, fried/roast potatoes	≥6/wk	≤3/wk
Wine	–	white wine, red wine, fortified wine	≥4/d for men, ≥2/d for women	1.5-2.5/d for men, 0.5-1.5/d for women
Sweets	sweet biscuits, cakes buns pastries, sugar confectionery, chocolate confectionery, ice cream lollies, puddings and fruit pies, milk puddings	added sugars & preserves, chocolate confectionery, other sweets, biscuits, milk-dairy desserts, other desserts & cakes & pastries	≥4/wk	≤2/wk
Fat	–	olive oil	Non-consumers	Consumers

A moderate intake of fruits, nuts, cereals, dairy, white meat, and eggs was recommended. Points were allocated continuously between 0 (no consumption) and 1 (achieving an intake within the recommended level). Overconsumption (consuming an amount double the mid-point of the recommended intake), was penalised and received a maximum of 0.5 points, with points allocated proportionally between the recommended level and the penalty point.

Table S2. Variables for the multiple imputation by chained equations

Variable	Type of variable	Description of variable	Model used to predict missing data in this variable	N (%) with data on this variable
**Outcome variables**				
PyrMDS, age 46 years	Continuous	Range 0-15, with a higher score representing greater adherence to the Mediterranean diet pyramid	Linear regression	5943 (48%)
**Exposure variable**				
Early adulthood SETs, age 16-24 years	Categorical	Six classes of Continued Education, Managerial Employment, Skilled Non-manual Employment, Skilled Manual Employment, Partly Skilled Employment, and Economically Inactive	Not appliable	12423 (100%)
**Mediators**				
Household equivalent income, age 46 years	Continuous	The weekly household income divided by (1 + 0.5 x number of additional adults + 0.3 x number of children aged 0-15 years)	Linear regression	7266 (58%)
Neighbourhood deprivation, age 46 years	Ordinal	Decile values of the Index of Multiple Deprivation Score 2015	Ordered logistic regression	7923 (64%)
**Covariates**				
Sex	Binary	Men and women	Not appliable	12423 (100%)
PyrMDS, age 16 years	Continuous	Range 0-13, with a higher score representing greater adherence to the Mediterranean diet pyramid	Linear regression	4079 (33%)
Exercise frequency, age 16 years	Ordinal	0, 0-3, 3-5, >5 times/week of playing sports more than 30 minutes	Ordered logistic regression	4571 (37%)
Sleeping hours, age 16 years	Continuous	Hours spent for sleeping in the previous day of the survey	Linear regression	4437 (36%)
Smoking frequency, age 16 years	Ordinal	None, less than one, one to four, and 5 or more cigarettes/week	Ordered logistic regression	4760 (38%)
Alcohol use, age 16 years	Continuous	Range 0-7, number of days having alcohol in the past week of the survey	Linear regression	4284 (34%)
General health, age 16 years	Ordinal	Excellent, good, fair, and poor	Ordered logistic regression	7052 (57%)
Malaise scale, age 16 years	Continuous	Range 0-22, with a higher score representing experiencing more symptoms associated with depression	Linear regression	4760 (38%)
BMI, age 16 years	Continuous	Parental report of weight (kg) divided by the square of parental report of height (metres)	Linear regression	4975 (40%)
Parental social class, age 10 years	Ordinal	Professional, managerial, skilled non-manual, skilled manual, partly skilled, and unskilled	Ordered logistic regression	10344 (83%)
Parental education, age 10 years	Ordinal	No qualifications, trade apprenticeship/O-level, A-level, further vocational, and degree+	Ordered logistic regression	10490 (84%)
Household income, age 10 years	Ordinal	>250, 200-250, 150-200, 100-150, 50-100, 35-50, <35 pounds/week	Ordered logistic regression	9824 (79%)
Family structure, age 10 years	Categorical	living with both parents, with one of the parents, and with others	Polytomous logistic regression	10682 (86%)
Neighbourhood location, age 10 years	Categorical	Rural, country-in or close to village, outskirts or town or city, inner urban area, and council estate	Polytomous logistic regression	10227 (82%)
Neighbourhood social rating, age 5 years	Ordinal	Range from 1 to 4, with a higher level representing a more well-to-do neighbourhood	Ordered logistic regression	9826 (79%)
**Auxiliary variables for outcomes**				

Days of having breakfast, age 42 years	Continuous	Range 0-7	Linear regression	7986 (64%)
Frequency of having convenience food, age 42 years	Ordinal	More than once a day, once a day, several times a week, once or twice a week, at least once a month, less often, and never	Ordered logistic regression	8068 (65%)
Intake of fruit and vegetables, age 30 years	Ordinal	Servings of fruit and vegetables a day	Ordered logistic regression	11153 (90%)
**Auxiliary variables for mediators**				
NSSEC8, age 42 years	Ordinal	Range 1-8, from higher managerial, administrative and professional occupations to never worked and long-term unemployed	Ordered logistic regression	8005 (64%)
Cohort member and partner's take home income, age 42 years	Ordinal	Range 1-18, with a higher level representing higher take home income from all sources	Ordered logistic regression	6277 (51%)
**Auxiliary variables for diet**				
Intake of soft drink, age 10 years	Binary	Having soft drink every day, and less often	Binary logistic regression	9890 (80%)
Intake of sweets, age 10 years	Ordinal	Nearly every day, quite often, sometimes, and hardly ever	Ordered logistic regression	9890 (80%)
**Auxiliary variables for health covariates**				
Frequency of playing sports, age 10 years	Ordinal	Never or hardly ever, sometimes, and often	Ordered logistic regression	10623 (86%)
Sleep difficulty, age 10 years	Binary	Having sleep difficulty, and not having sleep difficulty	Binary logistic regression	10654 (86%)
Smoking history, age 10 years	Binary	Having tried a cigarette, and never trying a cigarette	Binary logistic regression	9828 (79%)
BMI, age 10 years	Continuous	Parental report of weight (kg) divided by the square of parental report of height (metres)	Linear regression	9606 (77%)
Rutter behaviour scale, age 10 years	Ordinal	0-80th, 81st-95th, and >95th centile	Ordered logistic regression	10056 (81%)
Malaise Inventory score, age 26 years	Continuous	Range 0-24, with a higher score representing experiencing more symptoms associated with depression	Linear regression	8355 (67%)
**Auxiliary variables for SEP covariates**				
Father's social class, birth	Ordinal	Range from 1 to 6: SC 1, SC 2, SC3 non-manual, SC 3 manual, SC4, SC5	Ordered logistic regression	10719 (86%)
Mother's social class, birth	Ordinal	Range from 1 to 6: SC 1, SC 2, SC3 non-manual, SC 3 manual, SC4, SC5	Ordered logistic regression	10480 (84%)
Father's age at completion of education, birth	Continuous	Age in years	Linear regression	11005 (89%)
Mother's age at completion of education, birth	Continuous	Age in years	Linear regression	11398 (92%)
Family structure, age 5 years	Categorical	living with both parents, with one of the parents, and with others	Polytomous logistic regression	10150 (82%)

Note. To assist the imputation, we included the auxiliary variables as SEP at birth and at age 42 years, additional variables on diet and eating behaviours at ages 16, 30 and 42 years, and health variables at age 10 years.

Table S3. OLS regression results for the associations of early adulthood socioeconomic trajectories with adult diet quality at age 46 years, with sequential adjustment of covariates (n=12423)

	Estimated values of PyrMDS, β (95% CI)			
	Model 1	Model 2	Model 3	Model 4
Early adulthood SET classes				
Continued education	Reference	Reference	Reference	Reference
Managerial	-0.57 (-0.69, -0.44)	-0.55 (-0.67, -0.43)	-0.48 (-0.61, -0.35)	-0.47 (-0.60, -0.34)
Skilled non- manual	-0.62 (-0.74, -0.50)	-0.69 (-0.80, -0.57)	-0.59 (-0.71, -0.47)	-0.58 (-0.70, -0.46)
Skilled manual	-0.91 (-1.05, -0.77)	-0.78 (-0.93, -0.64)	-0.66 (-0.82, -0.50)	-0.64 (-0.81, -0.48)
Partly skilled	-0.94 (-1.08, -0.79)	-0.84 (-0.99, -0.69)	-0.70 (-0.86, -0.54)	-0.70 (-0.87, -0.54)
Economically inactive	-0.86 (-1.04, -0.69)	-0.90 (-1.11, -0.70)	-0.74 (-0.96, -0.53)	-0.73 (-0.94, -0.51)

Note. Model 1 is the unadjusted model, model 2 added the covariates of sex and adolescent health, model 3 further added the covariates of childhood SEP, and model 4, the fully adjusted model, added the covariate of adolescent diet quality.

Table S4. Modelled mean values (with 95% CI) of Mediterranean diet scores at age 46 by early adulthood SETs, only including participants who have data on the diet outcome at age 46y (n=5943)

	Mediterranean diet scores	
Early adulthood SETs	Mean	95% CI
Continued education	6.59	(6.45, 6.73)
Managerial	6.14	(5.99, 6.29)
Skilled non-manual	6.02	(5.88, 6.17)
Skilled manual	5.96	(5.81, 6.12)
Partly skilled	5.96	(5.80, 5.12)
Economically inactive	5.92	(5.70, 6.14)

Results are adjusted for the covariates of sex, childhood SEP (age 10 years), baseline Mediterranean diet (age 16 years), and baseline health-related behaviours and health status (age 16 years).
